# Vagus Nerve Stimulation as a Gateway to Interoception

**DOI:** 10.3389/fpsyg.2020.01659

**Published:** 2020-07-29

**Authors:** Albertyna Paciorek, Lina Skora

**Affiliations:** ^1^Faculty of Psychology, University of Warsaw, Warsaw, Poland; ^2^Sackler Centre for Consciousness Science, University of Sussex, Brighton, United Kingdom; ^3^School of Psychology, University of Sussex, Brighton, United Kingdom

**Keywords:** interoception, vagus nerve, vagus nerve stimulation, VNS, tVNS, transcutaneous vagus nerve stimulation

## Abstract

The last two decades have seen a growing interest in the study of interoception. Interoception can be understood as a hierarchical phenomenon, referring to the body-to-brain communication of internal signals, their sensing, encoding, and representation in the brain, influence on other cognitive and affective processes, and their conscious perception. Interoceptive signals have been notoriously challenging to manipulate in experimental settings. Here, we propose that this can be achieved through electrical stimulation of the vagus nerve (either in an invasive or non-invasive fashion). The vagus nerve is the main pathway for conveying information about the internal condition of the body to the brain. Despite its intrinsic involvement in interoception, surprisingly little research in the field has used Vagus Nerve Stimulation to explicitly modulate bodily signals. Here, we review a range of cognitive, affective and clinical research using Vagus Nerve Stimulation, showing that it can be applied to the study of interoception at each level of its hierarchy. This could have considerable implications for our understanding of the interoceptive dimension of cognition and affect in both health and disease, and lead to development of new therapeutic tools.

## Introduction

Interoception pertains to receiving, encoding, and representation of internal bodily signals in the brain, as well as their perception (Cameron, 2001; Craig, 2002; Critchley et al., 2004). Through interoception we know when our heart is beating fast, when we need to take a deep breath, and when we are hungry, thirsty, hot, cold, nauseous, tired, or alert. It encompasses both the non-conscious bodily signals themselves and our conscious perception of them. Growing research has shown interoception not only to be crucial for homeostasis and allostasis (acute change to achieve homeostasis), but also central in a range of cognitive and emotional processes, including memory, decision-making, emotional processing, social interactions, and even consciousness, body ownership and a sense of self (Critchley et al., 2001; Dunn et al., 2010; Shah et al., 2017; Berntson et al., 2018; Critchley and Garfinkel, 2018). By their nature, internal bodily processes are notoriously difficult to manipulate in experimental settings. The vagus nerve, the main cranial nerve in the human body known to be central in relaying visceral signals to the brain, is naturally implicated in interoception (Critchley and Harrison, 2013; Quadt et al., 2019; Yoris et al., 2019). Yet surprisingly little research in this area has used vagus nerve stimulation (VNS) to modulate bodily signaling. So far, to the best of our knowledge, only one recent study has explicitly related VNS to interoception (Villani et al., 2019). Here, we review the accumulated cognitive and clinical research on VNS and propose that this technique can indeed be used to modulate a wide range of interoception-related processes.

The vagus nerve (cranial nerve X) is the longest and one of the most widely distributed nerves in the body (*vagus* in Latin means “wandering”). As part of the parasympathetic division of the autonomic nervous system, the vagus nerve conveys information between visceral organs (i.e., organs located in the thoracic and abdominal cavities, including the heart) and the brain (Berthoud and Neuhuber, 2000; Craig, 2002; Critchley and Harrison, 2013). About 80% of the vagus nerve is composed of afferent fibers, projecting to the nucleus tractus solitarii (NTS) in the medulla, before being relayed further to other brainstem nuclei (broadly implicated in homeostatic control) and higher-order structures, including the thalamus, hippocampus, amygdala, and insula (Goehler et al., 2000; Saper, 2002). VNS involves electrical stimulation of the afferent fibers of the vagus nerve, either in an invasive variant (where the stimulator is implanted in the patient’s body) or a non-invasive variant where the stimulation is delivered transcutaneously through the auricular branch of the vagus nerve (tVNS, aVNS, or taVNS). Both VNS and tVNS reliably induce activation in the same brain areas (Narayanan et al., 2002; Frangos et al., 2015; Badran et al., 2018a). These areas, and their projections, and especially the insula, have been shown to be implicated in interoception (Craig, 2002).

Interoception has been conceptually divided into hierarchical levels (Garfinkel et al., 2015; Quadt et al., 2018), from low-level visceral afferents and their preconscious effects on cognitive or affective processes to psychological dimensions and metacognitive awareness of internal bodily processes. Because the vagus nerve is the main pathway relaying visceral signals into the brain, we propose that (t)VNS could affect interoception at each of those levels. Recent years have also seen a growing interest in the role of interoception in mental illness, proposing that interoceptive dysfunction can contribute to impairments in social, cognitive, behavioral, affective, and somatic processes associated with certain psychopathologies such as anxiety, depression, eating disorders, addiction, or post-traumatic stress disorder (PTSD) (Paulus and Stein, 2010; Harshaw, 2015; Khalsa et al., 2018). Those dysfunctions can occur at any level of interoceptive processing. Proposed mechanisms include aberrant processing of the afferent signal (e.g., noisy inputs) or weight given to its representations (e.g., overweighted interoceptive signal affecting subsequent evaluation of affective stimuli), abnormal expectations about bodily states, attentional or cognitive biases (e.g., hypervigilance to bodily states), or psychological biases (e.g., weak insight into one’s bodily state and its relevance to environmental context) (Khalsa et al., 2018; Petzschner et al., 2017; Gu et al., 2019).

This paper provides an overview of the research on the effects of VNS on interoception-related processes (both cognitive and clinical), from the lowest to highest level, and relates those findings to embodied models of brain function.

## Low-Level Afferent Interoceptive Signal

The low-level visceral signal, such as baroreceptor activity or blood oxygenation, is not consciously perceived unless there is a problem. However, according to embodied, interoception-based accounts of the conscious self, access to and evaluation of the signal from the physical body is necessary for the conscious “self” to arise in the first place (Seth et al., 2012; Seth, 2013; Cleeremans et al., 2020). Stimulated NTS, the projection area of the vagus nerve, activates the dorsal raphe and other areas known to control alertness (George et al., 2000). For this reason, VNS was proposed as a fruitful therapy for disorders of sleep or even consciousness (George et al., 2000; Naritoku et al., 2003), and was subsequently shown to promote improved sleep in rats (Rong et al., 2019) and human adults (Bretherton et al., 2019). Impressively Corazzol et al. (2017), in a single case study, demonstrated that long-term VNS may improve patient condition even after years in persistent unresponsive wakefulness (vegetative state), warranting future research in this area. This finding corroborates the proposal that a sense of conscious self is embodied – the experience of owning and identifying with one’s own body, and the experience of first-person perspective, are directly associated with multi-level representations of physiological condition of the body (Seth et al., 2012; Seth, 2013; Cleeremans et al., 2020). As such, (t)VNS may play a pivotal role in helping to restore this conscious, embodied sensation. This hypothesis could be further reinforced if classical paradigms exploring the sense of self, such as the rubber-hand or full-body illusion or sense of agency, proved to be modulated by (t)VNS.

In the cognitive literature, a demonstration that VNS directly manipulates the read-outs of basic visceral afferent signals has yet to be given. The most promising candidate is the modulation of Heartbeat Evoked Potential (HEP) amplitude. The cardiac signal is one of the most widely explored low-level afferent signals, reflected in electroencephalography as an event-related potential synchronized with cardiac R-peaks. HEP has been shown to predict individual heartbeat perception (Pollatos et al., 2005; Terhaar et al., 2012), and was proposed to reflect interoceptive belief updating (Ainley et al., 2016) and affective predictions (Gentsch et al., 2018; Marshall et al., 2018). It has also been shown to be modulated by internal (vs. external) attentional focus, proposed to reflect interoceptive precision (Petzschner et al., 2019). The HEP has been shown to originate in the insula, the brain region assumed to be key for interoception (Park et al., 2017), and activated through (t)VNS (e.g., Badran et al., 2018a). Interestingly, Park et al. (2017) confirmed the HEP’s functional role in self-consciousness by demonstrating HEP modulations as a response to an experimentally-induced altered sense of self-identification (full-body illusion). This result supports the proposal that interoceptive information is a crucial substrate of the sense of self and body ownership (Sierra and David, 2011; Critchley and Harrison, 2013; Crucianelli et al., 2018). Additionally, the sense of body ownership has been shown to be impaired after insula lesions (Karnath, 2005; Gandola et al., 2012; Moro et al., 2016). Given that (t)VNS reliably activates the interoceptive network (including the insula), it could constitute a tool for manipulating low-level interoceptive signals and their read-outs, such as the HEP, for research into consciousness, sense of self and body ownership.

## Preconscious Impacts on Cognitive Processes

While the previous, lowest level of the interoceptive hierarchy was concerned with the mere communication and detection of bodily afferents, the second, preconscious level refers to the early stages of central processing and pertains to the impact the afferent visceral signals have on cognitive and affective processing. The cardiac signal in particular has been shown to play a part in emotion processing (Bechara and Naqvi, 2004). There is evidence that processing of emotional faces is enhanced at systole compared to diastole (Garfinkel and Critchley, 2016), and the learning of fearful face-name pairs is better at systole than at diastole (interestingly, just in people with heightened interoceptive ability, and not for happy or neutral faces; Pfeifer et al., 2017). Higher interoceptive ability (encompassing perception and confidence in one’s perception of bodily signals; see next section) correlates with recognition memory for emotional pictures (Pollatos and Schandry, 2008) and words (Werner et al., 2010). “Gut feeling” is commonly used to mean intuition, pointing to a popular sensation of visceral signals influencing decisions. Adaptive behavior and optimal decision-making have indeed been proposed to be supported, or even guided, by visceral signals, where the physiological state of the body provides a reference frame for homeostatic or motivational value of given choice options (Damasio, 1994; Bechara, 2004; Gu and FitzGerald, 2014; Maniscalco and Rinaman, 2018). Although the (t)VNS literature has not focused on interoception, studies have shown that this technique modulates similar interoceptive processes to those mentioned above. Emerging evidence suggests that tVNS can improve facial emotion recognition (Colzato et al., 2017a), though, interestingly, not from entire bodies (Sellaro et al., 2018). Elsewhere, preliminary experimental evidence suggests that VNS can improve decision-making on the Iowa Gambling Task (Martin et al., 2004).

Another example of a physical state influencing cognitive function is the phenomenon of memory enhancement during somatosensory arousal. Traumatic memories are remembered particularly vividly. This phenomenon is crucially reliant on vagal transmission of various neuromodulators (Flood and Morley, 1988; Williams and Jensen, 1993; Nogueira et al., 1994; Talley et al., 2002). VNS may induce a state similar to arousal, most likely linked to secretion of noradrenaline and acetylcholine in the brain, neurotransmitters known to mediate attention (Martino et al., 2007; Klinkenberg et al., 2011). Studies have shown that (t)VNS may improve declarative memory retention (Clark et al., 1999; Ghacibeh et al., 2006; Jacobs et al., 2015; Broncel et al., 2020; Giraudier et al., 2020), even in patients with Alzheimer’s Disease (Clark et al., 1999; Sjogren et al., 2002; Merrill et al., 2006). Other authors have found mixed results in single-session studies (see Vonck et al., 2014 for review), depending on the stimulation settings (0.5 mA being optimal). Clinical interventions have capitalized on this memory-enhancing effect of (t)VNS to strengthen the formation of adaptive memories and behaviors after brain damage, e.g., “targeted plasticity” interventions (Hays et al., 2013). Interestingly, there is mixed evidence whether long-term VNS may lead to general memory improvement, which was not observed in those undergoing chronic VNS treatment for epilepsy, but was in depression (Aaronson et al., 2013; Vonck et al., 2014). Vonck et al. (2014) point out that cognitive dysfunctions are inherent to clinical depression and known to improve with improvement of depressive symptoms. This ties in with accounts of depression linking it to malfunctioning interoceptive evaluation of bodily signals (Barrett and Simmons, 2015; Quadt et al., 2018). Chronic aberrant interoceptive processing has maladaptive allostatic consequences for dealing with stress and, in particular, inflammation, which frequently cooccurs with depression. Indeed (Howland, 2014) points that the relationship between depression and inflammation may be mediated by the vagus nerve.

Malfunctioning interoceptive evaluation of bodily signals is also implicated in anxiety and panic. It may result from distorted interoceptive learning, when a benign sensation is experienced in the context of an initial panic attack, resulting in rapid conditioning. Overriding such strongly conditioned responses remains a challenge for most therapies. Fear extinction, a removal of conditioned response, is a gold standard therapy for PTSD (Genheimer et al., 2017), yet for many it remains not entirely effective, calling for its enhancement, potentially with VNS or tVNS. So far, however, the results are mixed, with promising results of VNS on fear extinction in rats (Peña et al., 2013, 2014; Noble et al., 2017; Souza et al., 2019, 2020), and with varied success of tVNS in humans (Burger et al., 2016, 2017; Genheimer et al., 2017; Szeska et al., 2020), which may depend on particular stimulation parameters (Hansen, 2019).

## Psychological Dimensions: Interoceptive Accuracy, Sensibility, and Awareness

Psychological dimensions of interception refer to the conscious perception of bodily signals (Garfinkel et al., 2015). These dimensions have been conceptualized across three levels: interoceptive accuracy (referring to objective accuracy in perceiving a bodily signal, e.g., one’s own heartbeat), sensibility (subjective beliefs about the ability to perceive own bodily sensations), and awareness (the correspondence between accuracy and confidence, i.e., a metacognitive aspect of interoceptive ability). Interoceptive accuracy and awareness are typically quantified with performance on bodily signal perception tasks, such as heartbeat counting or detection tasks (e.g., Schandry, 1981). Those who perform well on heartbeat detection tasks also tend to experience greater arousal and higher HEP amplitudes for emotional pictures (Herbert et al., 2007). Performance on such tasks is shown to correlate with the intensity of one’s own emotions (Wiens et al., 2000), perception of others’ emotions (Terasawa et al., 2014), and decision-making (Werner et al., 2009; Kandasamy et al., 2016), although the causal relations are still unclear.

The role of tVNS on the psychological dimensions of interoception was directly tested by Villani et al. (2019). They found tVNS to improve participants’ ability to correctly identify (but not count) their own heartbeats (interoceptive accuracy). Furthermore, participants reported higher confidence in their decisions under tVNS, but this did not lead to enhanced interoceptive awareness. This promising result, in light of the range of phenomena shown to be improved in individuals with higher interoceptive accuracy outlined above, offers an avenue for further research.

Deficits in interoceptive processing at this level are related to poor processing or evaluation of own bodily signals (e.g., impaired insight into one’s internal state and its relevance to environmental context; e.g., Paulus and Stein, 2010; Petzschner et al., 2017), and can predict emotional and affective psychopathology. Individuals with alexithymia, an impairment in recognizing one’s emotions which often cooccurs with autism (ASC), show reduced interoceptive accuracy (Ernst et al., 2013), as well as reduced brain activation in the insular cortex (Bird et al., 2010). Evidence for a reduction in interoceptive accuracy in the autistic population is mixed (Quadt et al., 2018), and correlated with co-existing alexithymia (Shah et al., 2016). Interestingly, autistic individuals tend to have elevated confidence (sensibility) relative to their performance accuracy (Garfinkel et al., 2016). A similar discrepancy, termed “interoceptive trait prediction error,” has been found in anxiety disorders (Paulus and Stein, 2006). Direct interventions with tVNS have been proposed for autism (Engineer et al., 2017) and anxiety (George et al., 2008). tVNS has been shown to alleviate some symptoms of ASC in epileptic patients with this comorbidity (see Levy et al., 2010; Jin and Kong, 2017 for review), which we hypothesize to be modulated by improvement in their interpretative processing. More research is needed to correlate treatment-induced change in ASC symptomatology and interoceptive accuracy, sensibility, and awareness.

Aberrant performance on interoceptive tasks (usually lower accuracy) has been shown in eating disorders (ED), such as anorexia nervosa (Pollatos et al., 2008; Van den Bergh et al., 2017), along with impaired ability to differentiate hunger and satiety, and reduced response to emotional states (Fassino et al., 2004). Together these characteristics suggest weakened interoceptive processing. Vagus nerve stimulation is slowly being recognized as a potential treatment to regulate food craving (Wernicke et al., 1993; Boveja and Widhany, 2003) proposed that vagal signal suppression could be helpful in treating obesity, signal stimulation in anorexia, and intermittent stimulation in bulimia, though experimental research is still needed. Further work might also identify whether the regulation of food cravings leads to changes in performance on interoceptive tasks.

Finally, pain has also been described in terms of interoceptive processing (Craig, 2003; Khalsa et al., 2009), with the vagus nerve crucial in relaying somatosensory sensations. Pain is usually caused by the activation of nociceptors and nociceptive pathways (Meyer et al., 2006), but it is also known to occur without corresponding activity, and nociceptors can be active without the sensation of pain (e.g., lack of reported pain by soldiers during battle, despite severe injuries), and be modulated by psychological state (Melzack et al., 1982; Mariana von Mohr, 2019). Deficits in interoceptive accuracy have been reported in patients with fibromyalgia (Duschek et al., 2015), lower back pain (Mehling et al., 2013) and migraines (see (Lernia et al., 2016) for review). VNS has been shown to modulate the sensation of pain in fibromyalgia (Lange et al., 2011) and migraines (Barbanti et al., 2015). Pain perception has been also reported to be reduced in patients treated with VNS for depression (Borckardt et al., 2005). This points toward the conclusion that vagus nerve stimulation affects interoceptive processing of pain, though more research is needed to elucidate the causes of individual differences in response to treatment.

## Metacognitive Level

The final, metacognitive level represents an executive dimension. It refers to one’s ability to flexibly switch between attending to and utilizing interoceptive and exteroceptive information in an adaptive manner. Though direct tests on such tasks are yet to be done, there is promising evidence that tVNS facilitates attentional switching in conflict situations, such as a number version of the Simon task (Fischer et al., 2018), allows rapid attentional adaptation (Colzato et al., 2017b), and improves response selection in sequential action (Jongkees et al., 2018). In the clinical domain, tVNS has been shown to reduce temper outbursts in Prader-Willi Syndrome (Manning et al., 2019), further reinforcing the notion that it improves interoceptive processing at the high level implicated in executive control.

A VNS influence on metacognition may also account for the rare adverse side effect observed in 4 epilepsy patients, namely hallucinations and psychosis (De Herdt et al., 2003), most likely caused by acutely increased alertness and decreased sedation through VNS. Patients with mild or severe intellectual disability may be specifically prone to the development of these symptoms. We suggest that in cases where cognitive resources for the appraisal of peripheral, somatosensory information are impaired, augmenting the signal strength can lead to systemic overload and malfunction.

## Discussion

The range of processes which can be affected by stimulating the vagus nerve points to the sheer scale of visceral influence. The presented literature can be unified under the theoretical frameworks which posit that affective and cognitive states are continuously interpreted through, and biased by, the body’s internal states. While the primary function of interoceptive signals is to feed the brain a continuous stream of information on the internal state of the organism so as to ensure survival, there is increasing consensus that they also fundamentally inform motivational states, adaptive behavior and emotion (Damasio, 2010; Critchley and Harrison, 2013; Seth, 2013; Barrett and Simmons, 2015; Critchley and Garfinkel, 2018; Seth and Tsakiris, 2018). According to this view, the brain interprets its current environmental challenges in light of the concurrent state of the body. The evidence of VNS influence on higher-order processes can thus lend support to models of brain function that assume a causal visceral dimension.

The successful application of VNS in clinical settings, briefly outlined here in the context of interoception, also corroborates the idea that a number of clinical conditions may have an interoceptive dimension. The visceral contribution has been noted for anxiety, depression, and PTSD, and many of the conditions discussed above have overlapping symptoms (Khalsa et al., 2018). For example, (Howland, 2014) pointed out that the relationship between depression, inflammation, metabolic syndrome, and heart disease may be mediated by the vagus nerve. VNS research can lead to greater understanding of the interoceptive dimension in clinical conditions, giving rise to future treatments.

While VNS has been enjoying increasing clinical success and is a promising tool for interoceptive manipulation, it is noteworthy that the optimal experimental protocols are still a work in progress, and should be carefully considered on an individual basis. Considerations under ongoing debate include optimal stimulation locations on the ear (Burger and Verkuil, 2018), potential differences in signal paths between VNS and tVNS, and the longevity of the effect after stimulation ceases. It seems that although cognitive effects of VNS may be detectable after short periods of stimulation, even 20 min (e.g., Colzato et al., 2017b), attenuation of certain clinical symptoms may require much longer stimulation durations (e.g., Manning et al., 2019) point to reduction in number and severity of temper outbursts in Prader-Willi Syndrome with 4 h/daily stimulation (as recommended for epilepsy), applied for 6–9 months, but a prompt return of the symptoms in all 5 participants when the stimulation was subsequently reduced to 2 h. Researchers should also consider making age related adjustments, e.g., (Koo et al., 2001) point out that in children younger than 12 stimulation settings may require a higher stimulus current or longer pulse along with lower stimulus frequency than adults (e.g., stimulation at 20 Hz or lower instead of e.g., 30 Hz), as a child’s vagus nerve has slower conduction velocity. The effects of VNS on heart rate need to be considered as well. (Badran et al., 2018b) have shown that certain settings (pulse width 500 μs and frequency 10 Hz) are likely to lower the heart rate.

As for limitations of using VNS to modulate interoception, VNS cannot afford selective targeting of aspects of the interoceptive signal which may be of specific interest (e.g., heart, stomach, breath). As such, protocols should be designed to capitalize on VNS’s capacity to manipulate the strength of the afferent signal. Functional neuroimaging studies could be particularly amenable to this. For a thorough treatment of the limitations, see Kaniusas et al. (2019).

To conclude, vagus nerve stimulation affords a novel experimental approach to studying interoception and its role in cognitive and emotional processes and disorders at increasing levels of complexity. With developments in the method and optimal protocols gaining momentum, vagus nerve stimulation may prove a fruitful new gateway to interoception.

## Author Contributions

All authors listed have made a substantial, direct and intellectual contribution to the work, and approved it for publication.

## Conflict of Interest

The authors declare that the research was conducted in the absence of any commercial or financial relationships that could be construed as a potential conflict of interest.
